# Structural Polymorphism of Sorafenib Tosylate as a Key Factor in Its Solubility Differentiation

**DOI:** 10.3390/pharmaceutics13030384

**Published:** 2021-03-13

**Authors:** Gabriela Wiergowska, Anna Stasiłowicz, Andrzej Miklaszewski, Kornelia Lewandowska, Judyta Cielecka-Piontek

**Affiliations:** 1 Tarchomin Pharmaceutical Works “Polfa” S.A., A. Fleminga 2, 03-176 Warsaw, Poland; Gabriela.Wiergowska@polfa-tarchomin.com.pl; 2 Department of Pharmacognosy, Faculty of Pharmacy, Poznań University of Medical Sciences, Święcickiego 4, 60-781 Poznań, Poland; astasilowicz@ump.edu.pl; 3 Institute of Material Science and Engineering, Poznan University of Technology, Jana Pawła II 24, 60-965 Poznań, Poland; andrzej.miklaszewski@put.poznan.pl; 4 Institute of Molecular Physics, Polish Academy of Sciences, Smoluchowskiego 17, 60-179 Poznan, Poland; kornelia.lewandowska@ifmpan.poznan.pl

**Keywords:** sorafenib tosylate, polymorphic forms, solubility, permeability

## Abstract

The presence of active pharmaceutical ingredients (APIs) in the forms of different polymorphic states can induce differences in their physicochemical properties. In the case of poorly soluble APIs, like the oncological drug sorafenib tosylate, small variations in solubility may result in large bioavailability differences. The control of its therapeutic dose is crucial from the effective pharmacotherapy point of view and the reduction of side effects. Therefore, this study aimed to assess the influence of sorafenib tosylate polymorphic forms on its solubility and, consequently, permeability, based on passive diffusion through membranes simulating the gastrointestinal tract (GIT) conditions. In the first part of the work, two crystalline forms of sorafenib tosylate were identified using the X-ray powder diffraction, FT-IR, and Raman spectroscopy. Subsequently, solubility studies were carried out. Both forms of sorafenib tosylate were insoluble in 0.1 N hydrochloric acid (HCl), in acetate buffer (pH 4.5), and in phosphate buffer (pH 6.8). Solubility (mg/mL) of form I and III of sorafenib tosylate in 0.1 N HCl + 1.0% SDS was 0.314 ± 0.006 and 1.103 ± 0.014, respectively, in acetate buffer pH 4.5 + 1.0% SDS it was 2.404 ± 0.012 and 2.355 ± 0.009, respectively, and in phosphate buffer pH 6.8 + 1.0% SDS it was 0.051 ± 0.005 and 1.805 ± 0.023, respectively. The permeability study was assessed using the parallel artificial membrane permeability assay (PAMPA) model. The apparent permeability coefficient (*P_app_*_—_cm s^−1^) of form I and III in pH 1.2 was 3.01 × 10^−5^ ± 4.14 × 10^−7^ and 3.15 × 10^−5^ ± 1.89 × 10^−6^, respectively, while in pH 6.8 it was 2.72 × 10^−5^ ± 1.56 × 10^−6^ and 2.81 × 10^−5^ ± 9.0 × 10^−7^, respectively. Changes in sorafenib tosylate concentrations were determined by chromatography using the high-performance liquid chromatography (HPLC)–DAD technique. As a result of the research on the structural polymorphism of sorafenib tosylate, its full spectral characteristics and the possibility of using FT-IR and Raman spectroscopy for the study of polymorphic varieties were determined for the first time, and the HPLC method was developed, which is appropriate for the assessment of sorafenib solubility in various media. The consequences of various physicochemical properties resulting from differences in the solubility of sorafenib tosylate polymorphs are important for pre-formulation and formulation studies conducted with its participation and for the safety of oncological sorafenib therapy.

## 1. Introduction

Sorafenib is a multikinase inhibitor that inhibits mitogen-activated protein kinase (MAPK) pathway kinases rapidly accelerated fibrosarcoma (Raf)-1, wild-type B-Raf, and mutant B-Raf. The drug also inhibits vascular endothelial growth factor receptor (VEGFR)-1, VEGFR-2, VEGFR-3, platelet-derived growth factor receptor (PDGFR)-BETA, FMS-like tyrosine kinase 3 (FLT-3), receptor tyrosine kinase (c-KIT), and rearranged during transfection (RET) [1]. Sorafenib was reported to exist in a base form, in the forms of hydrochloride, hydrobromide, methylsulfonate, sulfate, hemi-tosylate and tosylate; however, only the last form is used in medicine [2]. Sorafenib tosylate is approved by the U.S. Food and Drug Administration (FDA) in the treatment of unresectable hepatocellular carcinoma; advanced renal cell carcinoma; and metastatic, radioactive iodine resistant, and differentiated thyroid carcinoma [3]. What is more, it is increasingly used off-label in acute myeloid leukemia. Sorafenib reduces tumor cell proliferation and angiogenesis. It is recommended to administer 400 mg of sorafenib in tablets orally two times daily without food or with low or moderate fat food [3].

The very low solubility of sorafenib tosylate places it in the drug group of poorly soluble compounds. The solubility of about 70% of the new pharmaceutical oral compounds in early states of research is less than 100 µg/mL and is often a limitation in achieving the required bioavailability. Sorafenib tosylate is insoluble in water, and therefore it is classified as class II in the Biopharmaceutics Classification System (BCS). Drugs from class II have high membrane permeability and poor solubility [4]. Unfortunately, oral bioavailability of sorafenib tablets (Nexavar) is moderate because it ranges from 38 to 49% [3]. Thus, changes in the bioavailability of sorafenib tosylate are possible due to changes in its solubility.

It is known that crystallographic polymorphism can determine differences in active pharmaceutical ingredient (API) solubility. For example, polymorphs of benzocaine, gliclazide, glibenclamide, tolbutamide, furosemide, cyclopenthiazide, piroxicam, tenoxicam, diflunisal, succinyl sulfathiazole, and fluprednisolone are characterized by different solubilities and ultimately different bioavailability [5]. That is why it is so important to define the solubility for each polymorph, including the evaluation of the dissolution rate changes in the case of amorphous dispersions of API. When obtaining various polymorphic and amorphous dispersion forms, in addition to physical stability, it is always necessary to control the chemical stability, which may change as a result of certain physical processes, for example those taking place during tableting [6,7]. To our knowledge, no results have been reported showing the effect of the polymorphic forms of sorafenib tosylate on its solubility. Sorafenib tosylate can exists in three polymorphic forms, namely thermodynamically stable form I, metastable form II, and form III, which can convert to form I, especially when it is contaminated with that form. High temperatures promote that conversion. Melting points of the forms are 223–231 °C, 194 °C, and 187–190 °C for form I, II, and III, respectively. The patents claim methods for the synthesis of further polymorphic forms of sorafenib tosylate [8,9]. The poor solubility of sorafenib is a significant limitation in its use, therefore research was conducted to search for solvents ensuring its required solubility. In the literature on the subject, there are reports about the possibility of obtaining various solvates of sorafenib tosylate from methanol, ethanol, and n-methyl-2-pyrrolidone. These solvates show different thermodynamic stability [10]. The solubility of sorafenib was studied in the form of a base as well as in salt in many solvents. The solubility of sorafenib in different monosolvents increases with temperature (283–333 K), while only in binary solvents (2-propanol and 1,4-dioxane) did it show the maximum [11]. The solubility of sorafenib tosylate in subcritical carbon dioxide was also investigated. Studies were performed in the temperature range of 308–333 K and 120–270 bar [12]. Yang Guo et al. confirmed that the preparation of the nanomatrix of sorafenib tosylate with mesoporous materials (Sylysia) improves antitumor activity due to better solubility in water [13].

The aim of the study was to investigate the effect of the crystalline form of sorafenib on its solubility and the dynamics of permeability, based on passive diffusion, through membranes simulating the walls of the gastrointestinal system. In the first part of the work, the identification of the studied crystal forms of sorafenib tosylate was performed. Then, the solubility of both polymorphic forms of sorafenib tosylate at a different pH was determined, and their permeability through the system of artificial biological membranes was determined.

## 2. Materials and methods

### 2.1. Materials

Sorafenib tosylate samples were provided by Cipla.com (accessed on 31 January 2021) (form I) and Qilu.com (form III). Form I of sorafenib tosylate is off-white powder and form III is white powder.

### 2.2. Characterization of Polymorphic Forms of Sorafenib Tosylate

The polymorphic forms of API were identified via X-ray powder diffraction studies (XRPD), Fourier-transform infrared (FT-IR) spectroscopy, and Raman spectroscopy, along with density-functional theory (DFT) calculations as supportive methods.

#### 2.2.1. X-ray Powder Diffraction

The identification of polymorphic forms of sorafenib I and III was carried out via XRPD and compared to reference data. Diffraction patterns were measured on a PANalytical Empyrean diffractometer (Malvern Panalytical, Malvern, UK) with CuKα radiation (1.54056 Å) at a tube voltage of 45 kV and a tube current of 40 mA. The angular range was 3° to 50° with a step size of 0.017° and counting rate of 15 s/step. OriginPro 8 was used to analyze the acquired data [14,15].

#### 2.2.2. FT-IR Spectroscopy

Spectra of sorafenib tosylate were recorded in a frequency range of 400–4000 cm^−1^ on an FT-IR Bruker Equinox 55 (Bruker, Billerica, MA, USA) spectrometer. The analysis of changes in experimental spectra of sorafenib was conducted based on the theoretical spectrum. All the calculations were performed by using the Gaussian 09 package [16]. The molecular geometries were optimized using the density functional theory (DFT) method with Becke’s three-parameter hybrid functional (B3LYP) implemented with the standard 6–311 G(d,p) as a basis set. The calculations of normal mode frequencies and intensities were also performed. All calculations were carried out using the Gaussian 09 package (Wallingford, CT, USA). The GaussView (Wallingford, CT, USA, Version E01) program was used to propose an initial geometry of investigated molecules and for visual inspection of the normal modes [17]. The scaling factor for vibrational calculations was 0.967.

#### 2.2.3. Raman Spectroscopy

Raman spectra of sorafenib samples were obtained with a LabRAM HR800 spectrometer (HORIBA Jobin Yvon, Montpellier, France) with laser (He–Ne) excitation of λ_exc_ = 633 nm. The spectra were recorded at the range 200–2000 cm^−1^.

### 2.3. Solubility and Permeability Studies of Polymorphic Forms of Sorafenib Tosylate

#### 2.3.1. High-Performance Liquid Chromatography (HPLC) Method

The HPLC reversed-phase assay was carried out using an octadecylsilica column (3 μm, 50 mm × 4.6 mm i.d.) at a temperature 40 °C. The isocratic mobile phase consisted of a mixture solution 0.02 M of sodium dihydrogen phosphate and acetonitrile (35:65, *v*/*v*) filtered through a membrane (0.45 μm) (Supplementary Table S2). The flow rate was 1.5 mL/min and the injection volume 5 μL. The detection wavelength was 266 nm and the retention time was 2 min. Selection of detection wavelength was based on maximum position of the most long-wavelength absorption band in the UV spectrum of sorafenib (266 nm). The chromatographic method was validated by simultaneous analysis of samples according to the International Conference on Harmonization (ICH) Q2(R2), Validation of Analytical Procedures: Definition and Methodology, considering validation parameters as specificity, linearity, and stability. Linearity regression data and the RSD (relative standard deviation) from response factors (ratios of peak areas and analyte concentrations) were determined and evaluated. The standard curve correlation coefficient was 0.997, and RSD from response factors was 1.2%. No interferences of sorafenib from solvents or filters in peak form were observed.

#### 2.3.2. Solubility Studies

The solubility of form I and III of sorafenib tosylate was performed in different media: 1 N hydrochloric acid (HCl), 1 N HCl + 1% sodium dodecyl sulfate (SDS), phosphate buffer 0.2 M, pH 4.5, phosphate buffer 0.2 M, pH 4.5 + 1% SDS, phosphate buffer 0.2 M, pH 6.8, and phosphate buffer 0.2 M, pH 6.8 + 1% SDS. Sodium dodecyl sulfate (SDS) as anionic surfactant was used. The media were prepared according to the European Pharmacopoeia [18]. Phosphate buffer solution at pH 4.5 was prepared by dissolution of 13.61 g of potassium dihydrogen phosphate in 750 mL water, then by adjusting the pH with 85% ortophosphoric acid and dilution to 1000 mL with water. The medium with 1% SDS was prepared by dissolution of 1 g SDS in 100 mL of phosphate buffer (pH 4.5). The solubility of sorafenib from the coprecipitate was determined by agitating excess coprecipitate in 20 mL of proposed medium in a tube in 37 °C for 4 h (previously determined to be adequate time for equilibration). After 4 h, 5 mL of solution was withdrawn and filtered through a 0.2 µm membrane filter. Then, 1 mL of filtrate was transferred to 10 mL volumetric flask and filled up to volume with solvent and analyzed by the HPLC method.

#### 2.3.3. Permeability Study

In vitro gastrointestinal (GIT) permeability was performed using PAMPA (parallel artificial membrane permeability assay). The sandwich consists of two plates, a 96-well microfilter plate and a 96-well filter plate. The system is divided into two chambers, the donor at the bottom and the acceptor at the top, separated by a 120-μm-thick microfilter disc coated with a 20% (*w*/*v*) dodecane solution of a lecithin mixture (Pion, Inc.). The samples were dissolved in dimethyl sulfoxide (DMSO), diluted in donor solution in a different 96-well filter plate, and added to the donor compartments. The donor solutions were adjusted to acidic and basic pH (1.2 and 6.8, respectively). The changes in concentrations of sorafenib tosylate in the compartments were measured using the HPLC–DAD method after 4 h of incubation at 37 °C and rotational speed of 50 rpm. The apparent permeability coefficient (*P*_app_) was calculated by using the following equation:

$$P_{app}=\frac{-\ln\left(1-\frac{C_{A}}{C_{equilibrium}}\right)}{S\times \left(\frac{1}{V_{D}}+\frac{1}{V_{A}}\right)\times t}$$

where *V_D_*—donor volume, *V_A_*—acceptor volume, *C_equilibrium_*—equilibrium concentration $C_{equilibrium}=\frac{C_{D}\times V_{D}+C_{A}\times V_{A}}{V_{D}+V_{A}}$, *S*—membrane area, *t*—incubation time (s). Substances with *P_app_* < 0.1 × 10^−6^ cm s^−1^ are referred as low permeable, while compounds with a medium permeability have a 0.1 × 10^−6^ cm s^−1^ ≤ *P_app_* < 1 × 10^−6^ cm s^−1^, and substances with a *P_app_* ≥ 1 × 10^−6^ cm s^−1^ are classified as high permeable [19].

## 3. Results and Discussion

Differentiation of different polymorphic forms of API might be based on instrumental techniques including XRD, differential scanning calorimetry, thermal gravimetric analysis, spectral analysis, Raman, infrared, solid-state nuclear magnetic resonance, and microscopy. Due to FDA recommendations, analysis of the diffractograms of the XRD technique should be used as definitive evidence of polymorphism [20]. However, it is valuable to compare the results with other techniques.

**Figure 1:**
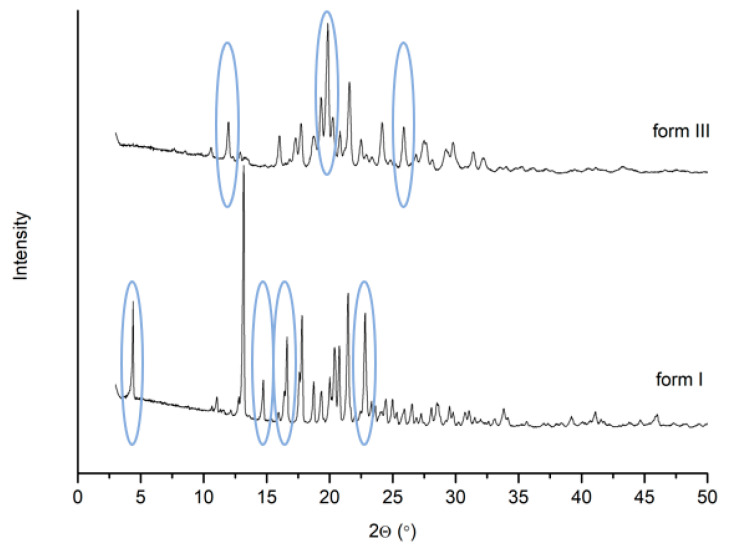
Diffractograms of sorafenib tosylate, form III and form I.

The analysis of the diffractograms of the two tested samples of sorafenib tosylate allowed for the identification of significant structural differences and to be able to classify them as polymorphic forms I and III [8,10,21] (Figure 1). The characteristic reflections for form I (4.4°, 14.8°, 16.7°, 22.9°) and for form III (12.0°, 19.9°, 25.9°) are marked.

**Figure 2:**
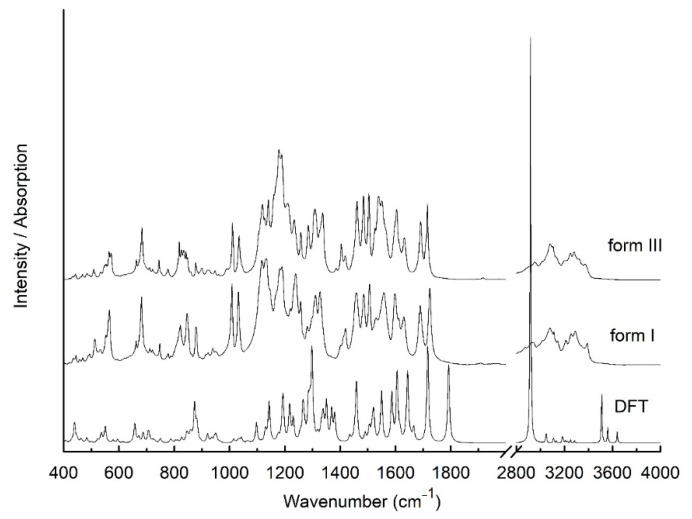
Calculation (DFT) and experimental IR absorption spectra of sorafenib tosylate form I and form III at room temperature.

**Figure 3:**
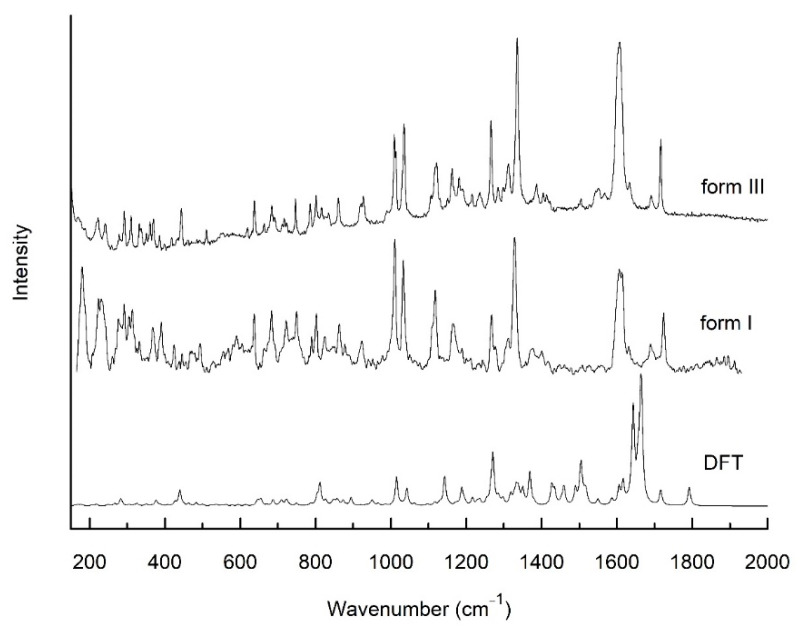
Calculation (DFT) and experimental Raman scattering spectra of sorafenib tosylate form I and form III at room temperature.

As a complementary to the XRPD differentiation of two polymorphs of sorafenib tosylate, we proposed FT-IR and Raman techniques (Figure 2 and Figure 3). Using theoretic calculations, we determined the theoretical spectrum of sorafenib tosylate—the reference spectrum. Based on the comparison with the reference spectrum, we described the characteristic bands; indicating the bands with changes allowed for samples to be distinguished from different polymorphic forms of sorafenib tosylate.

Most dissimilarities involved changing position of the bands, their shape, and relative intensity. In sorafenib tosylate we could distinguish 5 groups: 1—4-chloro-3-trifluoromethylphenyl, 2—ureido group, 3—phenoxy group, 4—pyridine-2-carboxylic-acid, and 5—methylamide-4-methylbenzene sulfonate. The greatest changes were visible in the bands associated with vibrations of the two external groups, 4-chloro-3-trifluoromethylphenyl and pyridine-2-carboxylic-acid. This is related to the presence of the methylamide-4-methylbenzene sulfonate group, which can interact with pyridine-2-carboxylic-acid through methylamide. The differences might occur in the region 800–900 cm^−1^, which, when observed, the bands were related to the vibration of the C–H deformation out of plane and S–O stretching bonds, and also in the region 1100–1350 cm^−1^, where the located bands were associated with the vibration of the C–S, C–F, C–O, C–N, and C–N–H stretching bonds. The band related to the bending vibration of the C–N–H bonds in methylamide located at about 1550 cm^−1^ was also changed. The characteristic bands of the first group, 4-chloro-3-trifluoromethylphenyl, were mainly related to the vibration of C–C, C–F, C–Cl, and C–H bonds. In the FT-IR spectra of form I and form III, the bands corresponding to the stretching vibration of the C–C bonds were located at 1032 and 1459 and 1034 and 1461 cm^−1^, respectively. The first band was also observed in Raman scattering spectra at 1030 and 1035 cm^−1^. The first band had an additional component related to the stretching vibration of the C–Cl bond, and the second was associated with the deformation of the C–N bond in the ureido group and stretching vibration of the C–F bonds. The bands related to the stretching vibration of the C–F bonds were also observed in FT-IR spectra at 1184 and 1190 cm^−1^ for form I, and 1177 and 1188 cm^−1^ for form III. In Raman scattering spectra, they were also visible at 1164 and 1186 cm^−1^ and 1161 and 1182 cm^−1^, respectively. In the range 650–950 cm^−1^, the bands were located corresponding to the deformation of the ring in the 4-chloro-3-trifluoromethylphenyl group; for instance, the bands at 681 cm^−1^ and 950/948 cm^−1^ for form I/form III. The last band was related to the deformation of the phenoxy ring and pyridine-2-carboxylic-acid. The strongest bands, which were observed in experimental IR absorption spectra at 1597 and 1629 cm^−1^ for SI, and at 1604 and 1632 cm^−1^ for form III, were related to the stretching vibration of the C=C bonds in the 4-chloro-3-trifluoromethylphenyl group, phenoxy ring, and pyridine-2-carboxylic-acid. They had additional components corresponding to the bending vibration of the C–N–H bonds in the ureido group. In Raman scattering spectra these bands were located at 1609 and 1929 cm^−1^ and 1606 and 1632 cm^−1^ for form I and form III, respectively. In the FT-IR and Raman scattering spectra, there were many bands related to the vibration of the bonds in the ureido group. In particular, there were the bands associated with the C–N stretching vibration. They were observed in FT-IR absorption spectra for SI at 1255, 1309, 1327, 1419, 1483, and 1528 cm^−1^, and for form III at 1260, 1309, 1338, 1420, 1483, and 1528 cm^−1^. The first three bands were also visible in Raman scattering spectra and were located for SI at 1268, 1310, and 1327 cm^−1^ and for form III at 1265, 1313, and 1336 cm^−1^. All of these bands also had additional components associated with C–N–H bending vibration, C–C and C–F stretching vibrations of the 4-chloro-3trifluoromethylphenyl group, or C–O stretching vibration of the ureido group. One of the most intensive bands in IR absorption spectra was related to the stretching vibration of the C=O bond in this group and was located at 1721 cm^−1^ for form I and at 1714 cm^−1^ for form III. In Raman spectra, it was located at 1723 and 1715 cm^−1^, respectively. At lower frequencies (711 cm^−1^), the observation was that the bands were related to the out of plane deformation vibration of the N–H bonds in the ureido group. For the phenoxy group, the observation was that the bands were associated with the stretching vibration of the C–C bond, which is described above. Furthermore, one more band was visible in IR absorption and Raman scattering spectra, at 1505/1506 for form I and 1502/1505 cm^−1^ for form III, which is related to the stretching vibration of the C–C bond. The characteristic bands of the fourth group also correspond to the stretching vibration of the C–C bonds; for example, there is the band that was about 1010 cm^−1^. These bands also had an additional component related to the stretching vibration of the C–N bond in pyridine-2-carboxylic-acid. Moreover, in IR absorption spectra, two bands were observed as being associated with the stretching vibration of the C–O and C=O bonds located for form I at 1218 and 1688 cm^−1^ and for form III at 1208 and 1691 cm^−1^. The second band was also observed in Raman scattering spectra, and it was located at 1688 and 1690 cm^−1^ for form I and form III, respectively. The band located in IR absorption spectra at 1279 for form I and at 1283 cm^−1^ for form III corresponded to the stretching vibration of the C–N bond pyridine-2-carboxylic-acid. For the last group—methylamide-4-methylbenzene sulfonate—the characteristic bands were related to the vibration of C–S and S=O. They were located, for example, at about 1115 cm^−1^. In IR absorption spectra at 877 for form I and 879 cm^−1^ was also observed the band corresponding to the stretching vibration of the S–O bonds. In this group were also observed the bands related to the bending vibration of the C–N–H bonds. One of them was visible in IR absorption spectra at 1556 for form I and 1550 cm^−1^ for form III. The second band was located in IR spectra at 1238 and 1234 cm^−1^ and in Raman scattering spectra at 1241 and 1238 cm^−1^ for form I and form III, respectively. These bands had additional components related to the stretching vibration of the C–O bond in pyridine-2-carboxylic-acid and the deformation vibration of the C–H bonds in the pyridine-2-carboxylic-acid and 4-chloro-3trifluoromethylphenyl groups. The bands related to out of plane deformation vibration of C–H bonds were also observed in the range 500–950 cm^−1^ (Supplementary Table S1). As described above, changes in the FT-IR and Raman spectra can be used to distinguish between structural polymorphs of sorafenib tosylate.

After confirming the two different structural forms of sorafenib tosylate, their solubility was assessed further in the study, also in the context of differences according to the structure (Table 1). The determination of the concentration changes was examined by liquid chromatography. The HPLC–DAD method was developed and validated (Supplementary Table S2, Figure S1). Sorafenib tosylate is insoluble in water; however, it is soluble in DMSO and ethanol. The effective way to impact the solubility is to change the medium by adjusting the pH or adding surfactants. Therefore, the solubility of polymorphic forms of sorafenib tosylate was tested in solvents with different pH values (0.1 N HCl—pH = 1; acetate buffer—pH = 4.5 and phosphate buffer—pH = 6.8). Sorafenib tosylate did not dissolve in any of the tested pHs. A significant improvement in the solubility of sorafenib tosylate was noted after adding 1% SDS to all solvents. For both structural forms, an improvement in sorafenib tosylate dissolution was observed upon dissolution in acetate buffer (pH = 4.5) after addition of 1% SDS. Interestingly, in the case of solvents with pH = 1.2 and pH > 6.0, the addition of 1% SDS resulted in significant changes in the solubility of the structural forms of sorafenib tosylate. However, form III turned out to be much more soluble.

As a consequence of solubility changes of the studied polymorphic forms of sorafenib tosylate, the dynamics of their penetration through membranes simulating the gastrointestinal walls was assessed using the PAMPA GIT model. The differences of permeability through an artificial membrane between samples of form I and III of sorafenib tosylate in acid and in a basic environment were studied (Table 2).

The form III of sorafenib tosylate had greater permeability in an acidic environment (3.15 × 10^−5^ ± 1.88 × 10^−6^ cm s^−1^) than form I (3.02 × 10^−5^ ± 4.13 × 10^−7^ cm s^−1^). The greater permeability in a basic environment was form III of sorafenib tosylate (2.81 × 10^−5^ ± 9.01 × 10^−7^ cm s^−1^) rather than form I (2.72 × 10^−5^ ± 1.56 × 10^−6^ cm s^−1^).

It is a necessity to monitor the occurrence of polymorphism among APIs, especially when their stability is low, and they can easily change from one form to another. Historically, we know cases of incidents involving a lack of polymorphism and control of drugs. An example of such a situation is polymorphic forms of ritonavir, which highly impact both the drugs solubility and dissolution rate [22]. In the beginning, due to a lack of polymorphism studies, soft gel capsules containing an ethanol/water solution of ritonavir were on the market. Afterward, thermodynamically stable form II was described, which has a 50% lower intrinsic solubility and precipitated out of solution, which forced the manufacturer to withdraw the product from the market. There are many examples in the literature of the influence of polymorphism on APIs. Carbamazepine’s form I and III, as well as dihydrate, differ in solubility. Form III is more soluble than form I and dihydrate, while the AUC value of form I is larger than AUC value of form III and dihydrate, which may result from the faster transformation of form III to dihydrate in gastrointestinal fluids, causing a reduction of dissolution rate [23]. Another study revealed that tadalafils polymorphic forms II, III, and IV show significantly lower dissolution and dissolution rates than the amorphous form of the API [24]. Polymorphic form II of tibolone is statically more soluble in water, 0.01 mol L^−1^ HCl and pH 4.5 acetate buffer. However, tibolone form II tablets have a lower release than tablets containing form I. These forms also differ in interactions with pharmaceutical excipients [25]. Our study confirms the importance of polymorphism studies and emphasizes the problem of varieties of physicochemical properties in different conditions.

## 4. Conclusions

As a result of the research on the identity of two polymorphic forms (I vs. III) sorafenib tosylate, it was confirmed that apart from changes in XRPD diffraction patterns, FT-IR and Raman analyses are valuable methods confirming the structural differences of the polymorphic forms.

Moreover, solvents were selected that ensure optimal solubility of the sparingly soluble sorafenib tosylate. Importantly, the research showed a significant influence of the crystalline structure of the polymorphs of sorafenib tosylate on their solubility and, consequently, on the dynamics of penetration, based on passive diffusion, through membranes simulating the walls of the gastrointestinal system.

## Figures and Tables

**Table 1:** Solubility of form I and form III of sorafenib tosylate in various media.

Medium	Solubility (mg/mL)	
	Form I	Form III
0.1 N HCl	-	-
0.1 N HCl + 1.0% SDS	0.314 ± 0.006	1.103 ± 0.014
Acetate buffer pH 4.5	-	-
Acetate buffer pH 4.5 + 1.0% SDS	2.404 ± 0.012	2.355 ± 0.009
Phosphate buffer pH 6.8	-	-
Phosphate buffer pH 6.8 + 1.0% SDS	0.051 ± 0.005	1.805 ± 0.023

**Table 2:** The permeability of form I and III of sorafenib tosylate through artificial membrane in acidic and basic pH.

pH	P_app_ (cm s^−1^)	
	Form I	Form III
1.2	3.01 × 10^−5^ ± 4.14 × 10^−7^	3.15 × 10^−5^ ± 1.89 × 10^−6^
6.8	2.72 × 10^−5^ ± 1.56 × 10^−6^	2.81 × 10^−5^ ± 9.0 × 10^−7^

## Data Availability

Data are available in a publicly accessible repository (see list of references).

## Supplementary Materials

The following are available online at https://www.mdpi.com/1999-4923/13/3/384/s1, Table S1.: Selected characteristic vibronic features of sorafenib tosylate in theory with application of 6–31 G(d,p) basis and experiment bands of sorafenib tosylate (def.—deformation, s—stretching, b—bending, oop—out of the plane); Table S2.: Parameters of liquid chromatography separation of sorafenib tosylate; Figure S1. Sorafenib tosylate chromatogram.

## Abbreviations

API	active pharmaceutical ingredient
BCS	Biopharmaceutics Classification System
c-KIT	receptor tyrosine kinase
DFT	density-functional theory
DMSO	dimethyl sulfoxide
FDA	Food and Drug Administration
FLT-3	FMS-like tyrosine kinase 3
FT-IR	Fourier-transform infrared spectroscopy
GIT	gastrointestinal tract
HCl	hydrochloric acid
HPLC	high-performance liquid chromatography
ICH	International Conference on Harmonization
MAPK	mitogen-activated protein kinase
PAMPA	parallel artificial membrane permeability assay
*P_app_*	permeability coefficient
PDGFR	platelet-derived growth factor receptor
RAF	rapidly accelerated fibrosarcoma
RET RSD	rearranged during transfection relative standard deviation
SDS	sodium dodecyl sulfate
VEGFR	vascular endothelial growth factor receptor
XRPD	X-ray powder diffraction
