# Supplementary material for: Structural Polymorphism of Sorafenib Tosylate as a Key Factor in Its Solubility Differentiation
Source: Pharmaceutics. 2021 Mar 13;13(3):384. doi: 10.3390/pharmaceutics13030384 (PMC8002161; doi:10.3390/pharmaceutics13030384)
Supplement: Supplementary file 1 [file pharmaceutics-13-00384-s001.pdf]

pharmaceutics

Supplementary Materials: Structural Polymorphism of Sorafenib Tosylate as a Key Factor in Its Solubility Differentiation

Gabriela Wiergowska, Anna Stasiłowicz, Andrzej Miklaszewski, Kornelia Lewandowska and Judyta Cielecka-Piontek *

Table S1. Selected characteristic vibronic features of sorafenib tosylate in theory with application of 6-31G(d,p) basis and experiment bands of sorafenib tosylate (def.-deformation, s-stretching, b-bending, oop out of the plane).

$\nu_{exp.R}$		$\nu_{exp.IR}$		$\nu_{t.}$	Bands assignment
S I	S III	S I	S III		
	509	512	508	535	C-H def. oop in methylobenzene sulfonate ring
		567	571	550	Def. methylobenzene sulfonate ring
662	664	661	664	657	C-S s + def. methylobenzene sulfonate ring
684	683	681	681	671	Def. 4-chloro-3trifluoro-methylphenyl ring
		711	711	687	N(1)-H def. oop in ureido
719	717	720	722	706	N(4)-H def. oop in in methylamide
	817	820	818	825	C-H def. oop
		846	846	846	C-H def. oop
		877	879	873	S-O s in methylobenzene sulfonate
921	928	920	922	920	C-H def. oop
		950	948	950	Def. 4-chloro-3trifluoro-methylphenyl, phenoxy and pyridine-2-carboxylic acid rings
1010	1009	1010	1010	1017	C-C s + C-N s in pyridine-2-carboxylic acid
1030	1035	1032	1034	1038	C-C s + C-Cl s in 4-chloro-3trifluoro-methylphenyl
921	928	920	922	920	C-H def. oop
		950	948	950	Def. 4-chloro-3trifluoro-methylphenyl, phenoxy and pyridine-2-carboxylic acid rings
1010	1009	1010	1010	1017	C-C s + C-N s in pyridine-2-carboxylic acid
1030	1035	1032	1034	1038	C-C s + C-Cl s in 4-chloro-3trifluoromethylphenyl
1115	1118	1117	1119	1143	C-S s + S=O s in methylobenzene sulfonate
1164	1161	1184	1177	1193	C-F s in 4-chloro-3trifluoro-methylphenyl

		950	948	950	Def. 4-chloro-3trifluoro-methylphenyl, phenoxy and pyridine-2-carboxylic acid rings
1010	1009	1010	1010	1017	C-C s + C-N s in pyridine-2-carboxylic acid
1030	1035	1032	1034	1038	C-C s + C-Cl s in 4-chloro-3tri-fluoromethylphenyl
1115	1118	1117	1119	1143	C-S s + S=O s in methylobenzene sulfonate
1164	1161	1184	1177	1193	C-F s in 4-chloro-3trifluoro-methylphenyl
1186	1182	1190	1188	1217	C-F s + C-H b in 4-chloro-3tri-fluoromethylphenyl and methylobenzene sulfonate
	1215	1218	1208	1230	C-O(2) s in pyridine-2-carboxylic acid + C-C s + C-H def. in phenoxy ring
1241	1238	1238	1234	1266	C-N(4)-H b in methylamine + C-O(2) s in pyridine-2-carboxylic acid + C-H def. in pyridine-2-carboxylic acid and 4-chloro-3tri-fluoromethylphenyl ,
1268	1265	1255	1260	1297	C-N s + C-N-H b in ureido + C-C s + C-F s in 4-chloro-3trifluoro-methylphenyl ,
	1285	1279	1283	1337	C-N(3) s + C-H def. in pyridine-2-carboxylic acid
1310	1313	1309	1309	1350	C-N(2) s C-O s in ureido + C-C s in 4-chloro-3trifluoromethylphenyl , phenoxy and pyridine-2-carboxylic acid
1327	1336	1327	1338	1369	C-N(1) s in ureido + C-C s + C-F s in 4-chloro-3trifluoro-methylphenyl
		1419	1420	1434	C-N(2)-H def. in ureido + C-C s + C-H def. in phenoxy ring
		1459	1461	1459	C-C s + C-N(1) def. + C-F s in in 4-chloro-3trifluoromethylphenyl and ureido
		1483	1483	1521	C-N(1) s + C-C s + C-H def. in ureido
1506	1505	1505	1502	1549	C-C s in phenoxy rings + C-H def. in phenoxy ring
		1528	1528	1586	C-N(1)-H b in ureido
	1551	1556	1550	1605	C-N(4)-H b in methylamide
1609	1606	1597	1604	1644	C=C s in 4-chloro-3trifluoro-methylphenyl and in pyridine-2-

					carboxylic acid + C-N-H b in ureido
1629	1632	1629	1632	1666	C=C s in 4-chloro-3trifluoro-methylphenyl and phenoxy rings + C-N(1)-H b
1688	1690	1688	1691	1716	C=O s in pyridine-2-carboxylic acid
1723	1715	1721	1714	1792	C=O s in ureido

Table S2. Parameters of liquid chromatography separation of sorafenib tosylate.

**Parameters**	**Determination of sorafenib tosylate**
Stationary phase (column):	Octadecylsilica column (3 µm, 50 mm × 4.6 mm i.d.)
Mobile phase:	A: 0.02 M of sodium dihydrogen phosphate (35%)<br>B: acetonitrile (65%)
Mobile Phase Flow Rate:	1.5 mL/min
Column temperature:	313K
Detection Wavelength:	266 nm
Injection volume:	5 µL

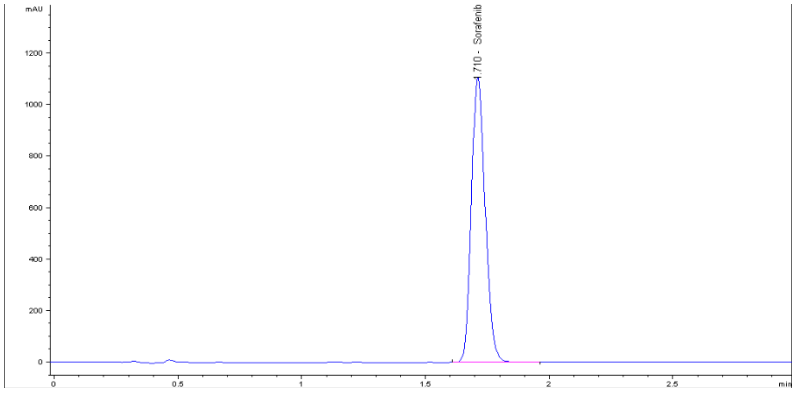

Figure S1. Sorafenib tosylate chromatogram.
